# Interface Bonding Behavior of Concrete-Filled Steel Tube Blended with Circulating Fluidized Bed Bottom Ash

**DOI:** 10.3390/ma14061529

**Published:** 2021-03-20

**Authors:** Lan Liu, Lei He, Zhi Cheng, Xiaoyi Wang, Zhe Ma, Xinrong Cheng

**Affiliations:** School of Science, North University of China, Taiyuan 030051, China; yunvlan@163.com (L.L.); 18434367366@163.com (L.H.); 18404912146@163.com (X.W.); mz605810831@163.com (Z.M.); 18234162820@163.com (X.C.)

**Keywords:** concrete-filled steel tube, circulating fluidized bed bottom ash, push-out test, bonding strength, bonding load

## Abstract

The interface bonding behavior between the steel tube and the concrete of concrete-filled steel tube (CFST) blended with circulating fluidized bed bottom ash (CFB-BA) was investigated in this study. A total of 8 groups of CFSTs stub columns were prepared with different dosage of CFB-BA, water-binder ratio (W/B), and interface bonding length. A series of push-out tests were carried out to acquire the data representing the interface bonding behavior. The results show that the dosage of CFB-BA has a direct effect on interface bonding behavior of CFST. CFB-BA can improve the interface bonding behavior of CFST. The highest ultimate bonding load and strength are achieved when the dosage of CFB-BA is 30%. When the dosage of CFB-BA increases to 50%, its interface bonding behavior decreases, but is still better than that of CFST without CFB-BA. W/B has a negative correlation with the interface bonding behavior of CFST. While the W/B increases, the interface bonding load and strength of CFST decreases. The increase of the interface bonding length can improve the interface bonding load, but cannot improve the interface bonding strength.

## 1. Introduction

Concrete-filled steel tube (CFST) is composed of steel tube and core concrete working together [1,2]. It has the characteristics of high bearing capacity, large lateral rigidity, good ductility and high construction efficiency [3,4,5,6,7,8]. CFST has been widely applied as the large load-bearing components in the construction because it can reduce the component size, save resources, speed up construction efficiency, and protect the environment [9,10].

The interface bonding force of the CFST consists of chemical bonding force, mechanical biting force, and frictional resistance [11]. There are many factors that affect the interface bonding strength of CFST, such as the shrinkage of concrete, water-to-binder ratio, and additives [12,13]. Xiushu Qu [14] studied the influence of factors such as the amount of concrete expansion agent and compressive strength of concrete on the bonding strength of concrete. The test results show that the compressive strength of concrete and the amount of expansion agent are the main factors affecting the interface bonding strength. Raed Abendeh [15] observed the bonding behavior by changing the material composition with rubber wire, and found that the shrinkage of concrete is also a vital factor affecting the bonding strength of CFSTs. The shrinkage of concrete can reduce the bonding strength of CFSTs, and even separate the steel tube and core concrete. Chang Xu [16] studied the interface bonding behavior of CFSTs with the expansive cement, and found that expansive cement can significantly enhance the bonding strength of CFSTs. The reduction of concrete shrinkage can improve the bonding strength of CFSTs.

Circulating fluidized bed bottom ash (CFB-BA) is a waste product from the bottom of circulating fluidized bed combustion in thermal power plants. CFB technology has been widely used due to its advantages such as good fuel adaptability, high efficient combustion, and low NO_x_ emissions [17,18,19]. The annual emissions of CFB-BA are very large, but its utilization rate is low [20]. Different from ordinary coal-fired fly ash, the particles of CFB-BA are loose and porous inside, so it has lager water absorption. Just like fly ash, CFB-BA also contain SiO_2_ and Al_2_O_3_, so it also has pozzolanic activity [21]. Researches have shown that CFB-BA has expansibility, which is closely related to the content of anhydrite and f-CaO [22,23,24]. To avoid the damage caused by expansion, the CFB-BA should be treated or the amount should be controlled during use. Mechanical grinding is a useful modified method for CFB-BA, which can improve the CFB-BA’s utilization and optimize its performance [22]. Zhi Cheng et al. [23] studied the expansion and load-deformation behavior of self-compacting concrete-filled steel tube stub column of CFB-BA. The results show that CFB-BA can improve the bearing capacity of concrete-filled steel tube stub columns.

The aforementioned works indicate that CFB-BA has expansibility, which is benificial for compensating the shrinkage of concrete. This paper presents the interface bonding behavior between the steel tube and the core concrete of CFST, which can contribute to the application of CFB-BA in CFST. In the paper, a controlled variable method was used to design a total of 8 groups of CFSTs, and push-out tests were carried out. The dosage of CFB-BA, the water-binder ratio and the interface bonding length are considered to be the main influencing factor. Load-slip curves, longitudinal strain distribution, and other data were obtained to analyze the interface bonding behavior of the CFSTs.

## 2. Experiment Part

### 2.1. Materials

**Figure 1:**
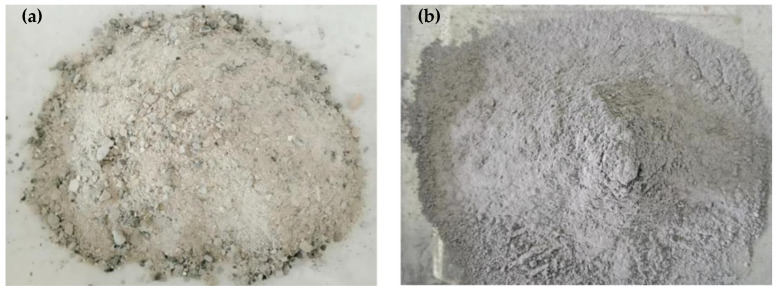
Digital images (**a**) Raw CFB-BA (**b**) Ground CFB-BA.

Grade P.O 42.5 Portland Cement (PC) (Chihoi, Taiyuan, China) was used in the experiment. The physical and mechanical properties of the PC are shown in Table 1. The CFB-BA from Pingshuo Coal Gangue Power Plant of Shuozhou, China was applied as well. After grinding with a ball mill for 39 min, it was passed through an 80 μm square hole sieve to gain a fine CFB-BAs. The main chemical compositions of CFB-BA are shown in Table 2. The ground CFB-BA was used as a mixture, which partly replaced PC. The surface area of the ground CFB-BA was 400 m^2^/kg. Particle size distribution of ground CFB-BA is shown in Table 3. Figure 1 shows the images of the raw CFB-BA and the ground CFB-BA.

The crushed stone (G) (5~20 mm) was used as the coarse aggregate, which meets the demand of the Chinese Industry Standard “GB/T 14685-2011 Pebble and crushed stone for construction” [25]. The fine aggregate was the natural sand (S) with a fineness module of 2.86–2.94, and the apparent density and bulk density were 2590 kg/m^3^ and 1550 kg/m^3^, respectively. The sand was sieved in compliance with the Chinese Industry Standard “GB/T 14684-2011 Construction sand” [26]. All aggregates were air-dried before use.

The polycarboxylate superplasticizer (PS) was from Baoding Muhu Hengyuan New Building Material Company (Baoding, China). Grade Q235 circular steel tubes with a straight slit (Tangshan Iron & Steel Group, Tangshan, China) were adopted to fabricate the test specimens. The steel tube was 2.5 mm in thickness and 89 mm in outer diameter. The material properties of steel are shown in Table 4.

### 2.2. Preparation of the Specimens

A total of 8 groups CFSTs were fabricated with a dosage of CFB-BA, W/B, and interface bonding length as variables. Each group contained two specimens, and the final test results were averaged accordingly. The self-compacting concretes (SCCs) were prepared, and the values of slump flow all exceed 650 mm. Before pouring the specimens, both ends of the steel tubes were polished, and the bottom was sealed with thin-film plastic to prevent the poured concrete from flowing out. The specimens were fabricated in the laboratory with a temperature of 20 ± 2 °C and a relative humidity of 50%. Three standard test cubes were reserved for each group to test the compressive strength of SCCs. The Mix proportions of SCCs is shown in Table 5, and the component parameters of CFSTs is shown in Table 6.

### 2.3. Test Setup and Instrument

**Figure 2:**
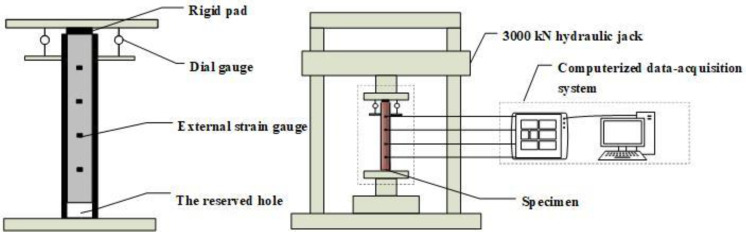
Test device model of CFST.

**Figure 3:**
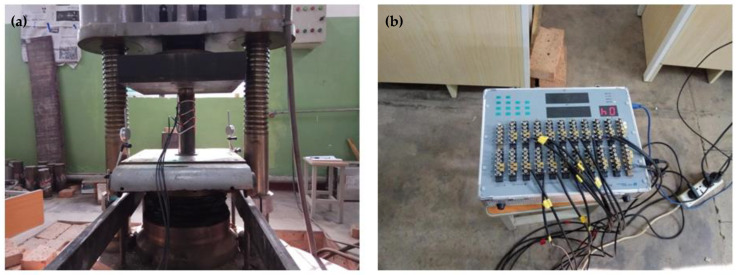
Test device system of CFST (**a**) loading system (**b**) data collection system.

**Figure 4:**
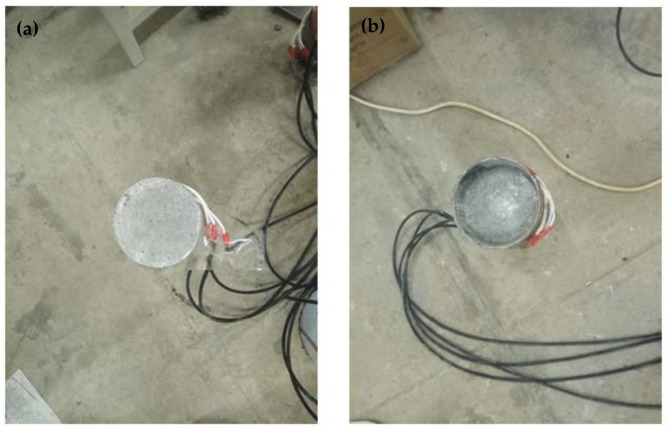
Comparison of CFST specimen (**a**) before loading (**b**) after loading.

The static grading load method was adopted in the push-out test. The test device model is shown in Figure 2. The steel tube at the free end of the test specimen was compressed. The core concrete at the loading end was also compressed. A rigid pad with a diameter slightly smaller than the steel tube was placed at the bottom of the core concrete at the loading end. Slippage occurred, and the core concrete was pushed out. Strain gauges were affixed every 50 mm in the longitudinal height of the steel tube specimen to measure the strain value along with the height. Two dial gauges were symmetrically arranged on the surface of the pressure plate to measure the relative slip between the steel tube and the concrete. Figure 3 shows the actual test device system, and Figure 4 shows the specimen before loading and after loading.

The loading process of the CFST specimen is divided into two stages, known as the pre-loading stage and formal loading stage.

During the process of test loading, the 5 kN load was pre-applied. The upper and lower pressure plates, specimens and pads of the press were in close contact so that the core concrete was evenly stressed.

In the formal loading stage, the load was graded, the rate was lower than 200 N/s, and the load was recorded every 5 kN load until the test specimens failed. At the beginning of loading, there was no change in the value displayed by the dial gauge and strain gauge. As the load increased, the loading process was gradually stabilized, and a small amount of slippage occurred at the end of the loading process. The relative amount of slippage became even larger as the load increased from the initial stage. The produce of the slippage means the chemical bonding force failure between the steel tube and the CFB-BA concrete [23]. In the later loading process, the load remained unchanged. The value of the dial gauge continued to increase, and the rigid pad has a pivotal displacement change, but the steel tube did not collapse. At this time, the bonding force at the interface of the CFST, and the specimens were broken. The test results of the specimens are shown in Table 7.

## 3. Results and Discussions

### 3.1. Load-Slip Curves of CFST

**Figure 5:**
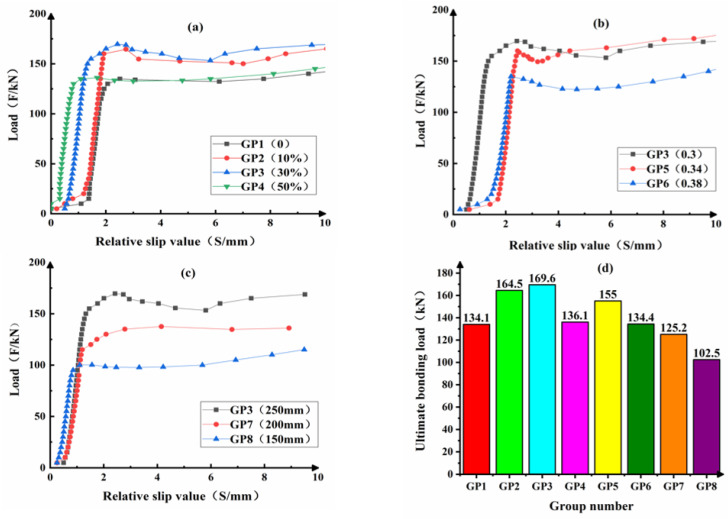
Load-slip curves of CFST (**a**) different dosage of CFB-BA (**b**) different W/B (**c**) different bonding length (**d**) ultimate bonding load.

The load-slip curves of CFST are shown in Figure 5. It can be seen that the shape of the load-slip curve of each group is similar to that of concrete-filled steel-tube without CFB-BA (GP1). In the entire process of interface adhesion from occurrence to destruction, the common characteristics can be concluded.

During the initial loading, slip occurs on a small scale between the concrete and the steel tube, because the core concrete is in direct contact with the loading slab. The core concrete produces the elastic compression deformation, causing slip afterward. As the load increases, the relative slip between the steel tube and the core concrete undergoes a process from slight slip to partial slip, and finally to full slip. When the load is increased to the failure load of the component interface bonding, the load-slip curve grows almost linearly. Besides, the interface bonding force is continuous along with the component length. With the increase of the load, the relative slip also increases with the speed lower than the load speed. When the applied load is close to the interface failure load, the relative curvature of the load-displacement curve becomes smaller. Load-slip curve shows an evident inflection point. With the increasing of component relative slip, the load has no significant change. The curve stays at a certain level for a period of time.

**Figure 6:**
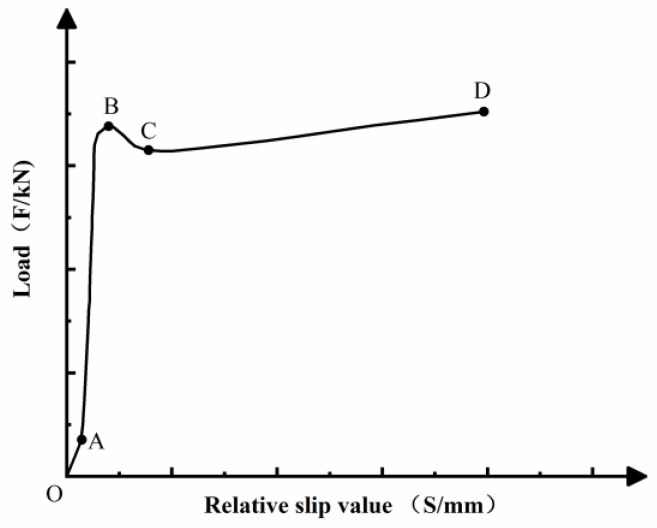
Load-slip curve model of CFST.

Figure 6 shows the load-slip curve model of CFST. As seen in Figure 6, the curve is consisted by four sections, which are OA, AB, BC and CD.

In the OA section, the initial load is rather small, and the specimens have a slight relative slip. At this time, the interface bonding force of the CFST is mainly borne by the chemical bonding force. The main factors affecting the chemical bonding force are cement dosage, concrete strength, and water binder ratio [23]. The chemical bonding force is small. When the applied load is low, an insignificant local relative slip occurs between the steel tube and the core concrete. At this time, the chemical bonding force of the part where the relative slip occurs has failed.

In the AB section, when the load continues increasing, a relatively large slip occurs between the core concrete and the steel tube. The local relative slip becomes more evident. At this time, the load on the specimen increases faster than the increase of relative slip. The interface bonding force is mainly caused by part of the chemical bonding force and mechanical biting force. When the chemical bonding force and mechanical biting force on the interface reach the maximum value, the load is near to the ultimate bonding load (*F*_U_). The load shows approximate linear distribution before the load is near to point B.

In the BC section, when the load reaches point B, the relative slip of the CFST interface enters a nonlinear phase. The core concrete has slipped to the steel tube. At this time, the chemical bonding force and mechanical bite force have failed. The interface bonding force is mainly borne by the friction between the steel tube and the core concrete. In this stage, even if a small load is applied, the displacement between the steel tube and the core concrete will be significantly changed.

In the CD section, as the slippage continues to develop, the concrete expands circumferentially under axial compression, and lateral extension produces. The concrete’s lateral extension results in the increase of the steel tube’s lateral pressure, which enlarges the sliding friction on the interface, so the CD section shows an upward trend.

### 3.2. Longitudinal Strain Distribution of CFST

**Figure 7:**
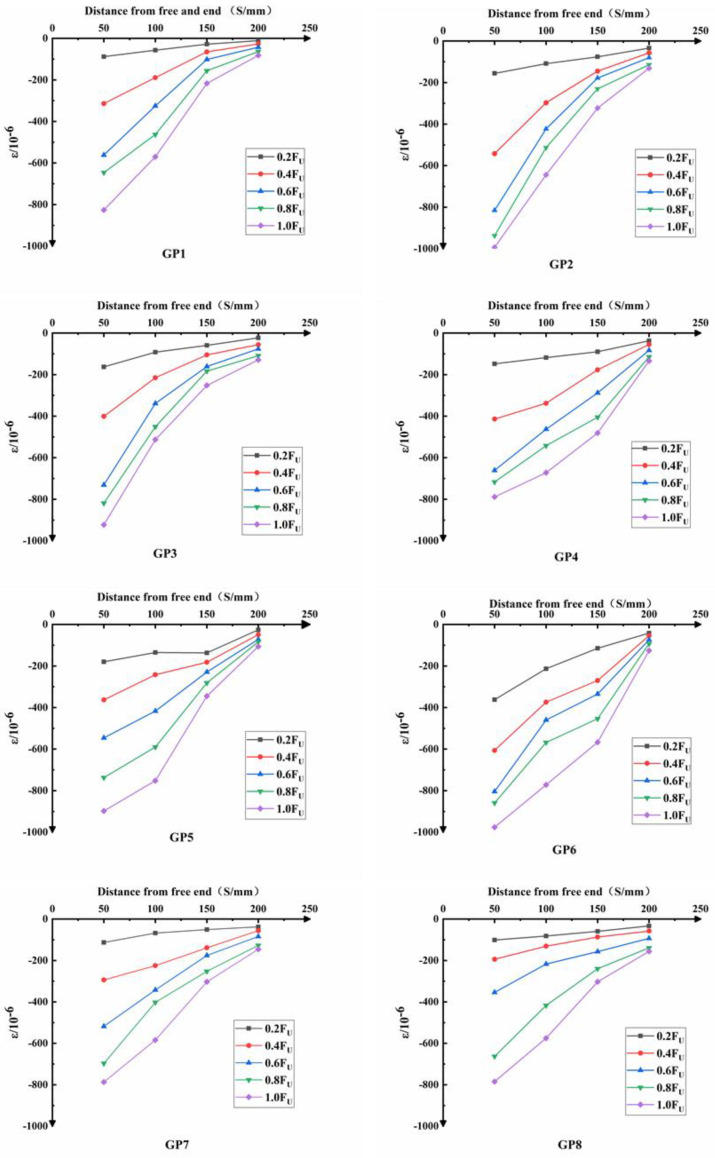
The curve of longitudinal strain distribution of CFST. Note: *F*_U_ means the ultimate bonding load.

The curve of longitudinal strain distribution of CFST is shown in Figure 7. It can be seen from Figure 7 that at the initial loading process, the longitudinal strain along the height of the steel tube shows no difference under the small load. The force transmission between the steel tube and the concrete in the specimens are relatively uniform. As the load gradually increases, the longitudinal strain along the height of the component also increases. Then, the continuity of the force transmission between the steel tube and the core concrete is gradually broke, which means that the bonding strength between the steel tube and the core concrete is damaged. A relative slip occurs between the steel tube and the core concrete. From the analysis on the strain curve of each specimen, it can be obtained that the largest longitudinal strain on the steel tube appears at its free end, and the smallest strain appears at its loading end.

Throughout the testing process, the steel tube was assumed to be always in the elastic stage before the specimen was damaged. In this test, the elastic modulus E of the steel tube is regarded as a fixed value. From the analysis of the stress state, when the core concrete in the steel tube is pushed out, it will produce a particular lateral squeeze on the steel tube with the action of thrust. The lateral squeeze makes the circular steel tube located in a circumferential tension state, causing a certain elastic deformation in the circumference of the steel tube. A small pushing load is applied to the test piece so that the core concrete can closely make contact with the steel tube. At this time, the core concrete exerted a specific lateral compression on the steel tube, which can increase the longitudinal strain of the steel tube. The core concrete is effectively restrained, which leads to an increase in the bonding stress. The increase of bonding stress is conducive to the cooperation of the steel tube and the concrete under external load.

### 3.3. Influence Factors on the Interface Bonding Behavior of CFST

**Figure 8:**
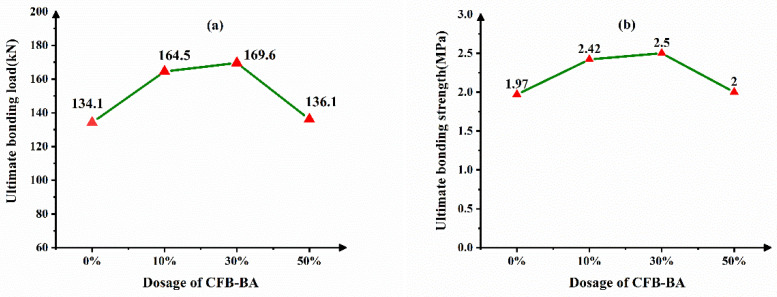
Dependence of CFB-BA dosage on the interface bonding behavior (**a**) ultimate bonding load (**b**) ultimate bonding strength.

Figure 8 shows the dependence of CFB-BA dosage on the interface bonding behavior. It can be seen from Figure 8 that when the amount of CFB-BA is 30%, the bonding load reaches the maximum. Comparing with the concrete filled steel tube without CFB-BA, its ultimate bonding load increased by 26.5%. The ultimate bonding loads of the concrete filled steel tube with 10% and 50% CFB-BA were higher than those without CFB-BA by 22.7% and 1.5%, respectively. The bonding strength of the group GP2 with 10% CFB-BA, the group GP3 with 30% CFB-BA, the group GP4 with 50% CFB-BA are 22.8%, 26.9%, 1.5%, respectively, which are all higher than that of the group GP1 without CFB-BA. CFB-BA contains Ⅱ-CaSO_4_ and f-CaO. In the hydration process of the cementitious system, f-CaO can hydrate to Ca(OH)_2_, and Ⅱ-CaSO_4_ can hydrate to gypsum. Gypsum can continue to react with Ca(OH)_2_ and hydrated calcium aluminate, and then produce ettringite. The ettringite can fill the pores and improve the compactness of the core concrete, and even produce self-stress in the CFST. So dosage of CFB-BA can improve the compressive strength of the core concrete, and make the core concrete and steel tube closer. But too much CFB-BA can lead to great expansion and anhydrite cannot hydrate completely, which decrease the compressive strength of the core concrete. So there exists a reasonable amount of CFB-BA. When the amount of CFB-BA is 30%, the CFST gains the maximum bonding load and maximum bonding strength [24].

**Figure 9:**
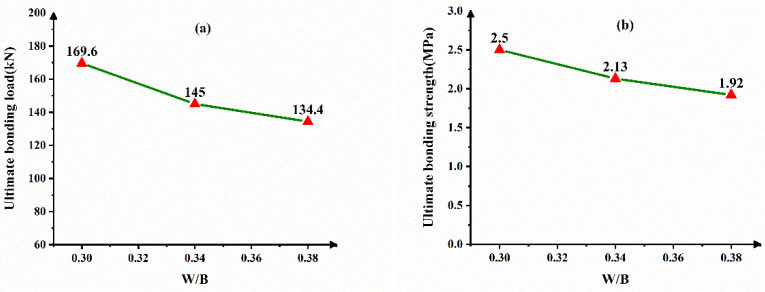
Dependence of W/B on the interface bonding behavior (**a**) ultimate bonding load (**b**) ultimate bonding strength.

Figure 9 shows the dependence of W/B on the interface bonding behavior. It can be seen from Figure 9 that when the amount of CFB-BA and the bonding length are fixed, the bonding load will reach the maximum with the water-cement ratio of 0.3. The bonding strength also reaches the maximum 2.50 MPa. With the increase of W/B, the bonding strength decreases by varying degrees. When the W/B is 0.34, the bonding strength is reduced by 14.8%. When the W/B is 0.38, the bonding strength is reduced by 23.2%. It can be seen from Table 6 that the measured compressive strength of the concrete cube decreases as the water-cement ratio increases. Cement mainly provides the cohesive force for the concrete. As the water-to-binder ratio increases, the actual amount of cement decreases, and the pores left by the sand and gravel aggregate cannot be filled. The porosity increases, the concrete is not dense, and the strength decreases. With higher water-cement ratio, the excess water may remain in the cement or evaporate to form pores or channels after the hardening process, resulting in a decrease in mechanical strength. The reduction in the strength of the core concrete will bring a decrease in the bonding strength of the CFST interface.

**Figure 10:**
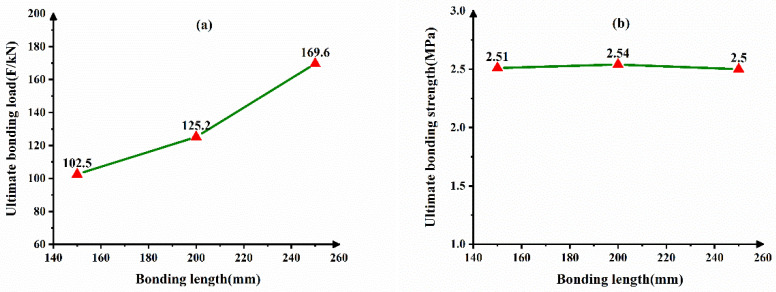
Dependence of bonding length on the interface bonding behavior (**a**) ultimate bonding load (**b**) ultimate bonding strength.

Figure 10 shows the dependence of bonding length on the interface bonding behavior. As shown in Figure 10, when the amount of CFB-BA and W/B is constant, the longer the interface bonding length, the higher the bonding load. However, the interface bonding strength shows no notable change. The reason is that, while the interface bonding length increases, the bonding force between the core concrete and the steel tube also increases. Thus, the increase of interface bonding length cannot improve the interface bonding strength.

## 4. Conclusions

The interface bonding behavior of CFST blended with CFB-BA was studied in the paper, and a total of 8 groups of CFST were designed for the push-out test. Several conclusions can be drawn as the following:

- The load slip curve of CFST blended with CFB-BA can be divided into four stages. The strains along the longitudinal height of the steel tube are the same as the ordinary CFST without CFB-BA.
- Adding CFB-BA within a certain range has a positive effect on the bonding strength of CFST. When the dosage of CFB-BA is 10%, 30% and 50%, the interface bonding strength of CFST increased by 22.8%, 26.9% and 1.5%, respectively. The micro-expansion property of the CFB-BA can make the concrete squeeze on the sidewall of the steel tube to improve the interface bonding strength. However, too much CFB-BA in concrete will reduce the strength of the concrete, thereby reducing the bonding strength of CFST.
- The W/B has an important influence on the interface bonding strength of CFST with CFB-BA. When the dosage of CFB-BA and the interface bonding length are constant, the higher the W/B, the lower the interface bonding strength of the CFST. W/B has negative correlation with the interface bonding load and bonding strength. When the W/B is 0.3, the interface bonding strength of the CFST reached the maximum, the value reaches 2.50 MPa.
- Bonding length has a significant effect on the ultimate bonding load of CFST. However, because the force-bearing area increases with the increase of the bonding length, the bonding strength of CFST is basically unaffected.

## Figures and Tables

**Table 1:** Properties of Portland Cement.

Setting Time (min)		Flexural Strength (MPa)		Compressive Strength (MPa)		Density (kg/m^3^)	Surface Area (m^2^/kg)
Initial	Final	3 d	28 d	3 d	28 d		
183	234	5.5	7.6	26.1	45.9	3100	350

**Table 2:** Main chemical compositions of CFB-BA (%).

SiO_2_	Al_2_O_3_	CaO	SO_3_	Fe_2_O_3_	MgO	K_2_O	P_2_O_5_	Na_2_O	Loss on Ignition
42.19	25.9	10.1	5.91	3.1	1.35	0.79	0.12	0.06	6.09

**Table 3:** Particle size distribution of ground CFB-BA.

Grinding Time (min)	Specific Surface Area (cm^2^/g)	X10 (µm)	X50 (µm)	X90 (µm)	X98 (µm)
39	400	1.40	15.07	63.86	97.23

**Table 4:** Properties of the steel tubes.

Thickness (mm)	Elastic Modulus (GPa)	Yield Strength (MPa)	Ultimate Strength (MPa)	Poisson’s Ratio
2.5	206	312	441	0.3

**Table 5:** Mix proportions of SCCs.

SCC	CFB-BA (%)	W/B	PC (kg)	CFB-BA (kg)	W (kg)	S (kg)	G (kg)	PS (%)
SCC-1	0	0	548.8	0.0	164.6	790.6	885.5	1
SCC-2	10	0.3	493.7	54.9	164.7	785.4	877.8	1.2
SCC-3	30	0.3	383.6	164.4	164.4	784.7	976.9	1.3
SCC-4	50	0.3	274.3	274.3	164.6	782.2	872.2	1.7
SCC-5	30	0.34	365.8	156.8	177.7	784.7	876.9	1.3
SCC-6	30	0.38	350.7	150.3	190.4	784.5	877.1	1.2

**Table 6:** Component parameters of CFSTs.

Group	SCC	CFB-BA (%)	W/B	Bonding Length (mm)	D × T × L (mm)
GP1	SCC-1	0	0.3	250	89 × 2.5 × 300
GP2	SCC-2	10	0.3	250	89 × 2.5 × 300
GP3	SCC-3	30	0.3	250	89 × 2.5 × 300
GP4	SCC-4	50	0.3	250	89 × 2.5 × 300
GP5	SCC-5	30	0.34	250	89 × 2.5 × 300
GP6	SCC-6	30	0.38	250	89 × 2.5 × 300
GP7	SCC-5	30	0.3	200	89 × 2.5 × 300
GP8	SCC-3	30	0.3	150	89 × 2.5 × 300

**Table 7:** Test results of specimens.

Group	CFB-BA (%)	W/B	Bonding Length (mm)	Compressive Strength at 28 d Age (MPa)	Ultimate Bonding Load (kN)	Ultimate Bonding Strength (MPa)	Relative Slip Value (mm)
GP1	0	0.3	250	57.1	134.1	1.97	2.514
GP2	10	0.3	250	52.4	164.5	2.42	2.486
GP3	30	0.3	250	55.5	169.6	2.50	2.286
GP4	50	0.3	250	48.8	136.1	2.00	1.200
GP5	30	0.34	250	46.6	155.0	2.13	2.199
GP6	30	0.38	250	37.1	134.4	1.92	2.097
GP7	30	0.3	200	55.5	125.2	2.54	1.573
GP8	30	0.3	150	55.5	102.5	2.51	1.017

## Data Availability

The data presented in this study are available on request from the corresponding author.
